# Clinical and Molecular Barriers to Understanding the Pathogenesis, Diagnosis, and Treatment of Complex Regional Pain Syndrome (CRPS)

**DOI:** 10.3390/ijms26062514

**Published:** 2025-03-11

**Authors:** Adam Zalewski, Iana Andreieva, Justyna Wiśniowska, Beata Tarnacka, Grażyna Gromadzka

**Affiliations:** 1 Department of Rehabilitation, Eleonora Reicher National Institute of Geriatrics, Rheumatology and Rehabilitation, Spartańska 1, 02-637 Warsaw, Poland; 2 Department of Rehabilitation Medicine, Faculty of Medicine, Warsaw Medical University, Spartańska 1, 02-637 Warsaw, Poland; andryana08@gmail.com; 3 Department of Biomedical Sciences, Faculty of Medicine, Collegium Medicum, Cardinal Stefan Wyszynski University in Warsaw, Wóycickiego Street 1/3, 01-938 Warsaw, Poland

**Keywords:** complex regional pain syndrome, algodystrophy, clinical, molecular, neuroinflammation, cytokines, microRNA, biomarkers, limitation

## Abstract

Complex regional pain syndrome (CRPS) is an idiopathic, highly debilitating chronic disorder with persistent regional pain accompanied by a combination of sensory, motor, and autonomic abnormalities. It is not only difficult to treat but also difficult to study. This scoping review aimed to identify the key clinical and molecular challenges encountered in CRPS research and to examine the assessment tools currently employed. A comprehensive search was conducted across PubMed/Medline, Science Direct, Scopus, Wiley Online Library, and Google Scholar using a combination of free text and MeSH terms related to CRPS, clinical and molecular aspects, neuroinflammation, biomarkers, and research challenges. We analyzed 55 original clinical research papers on CRPS and 17 studies of immunological/biochemical/molecular aspects of CRPS. A significant degree of heterogeneity was observed in the methodologies employed across the reviewed studies. The most frequently reported challenges included difficulties in participant recruitment and controlling confounding factors (reported in 62% of studies), such as the heterogeneity of the patient population, the influence of pain coping strategies and psychological factors, and the impact of sociocultural factors (reported in 62% of studies). Research into diagnostic and prognostic markers for CRPS also faces numerous challenges. Recruiting participants is difficult due to the rarity of the condition, resulting in small sample sizes for studies. In vitro models often fail to replicate the complexity of in vivo inflammation, limiting their applicability. Findings from early CRPS stages may not generalize to chronic CRPS because of differing pathophysiological mechanisms and symptom profiles. Additional obstacles include the disorder’s heterogeneity, difficulties in controlling confounding factors, variability in treatment approaches, and the lack of standardized tools and baseline comparisons. These issues hinder the development of reliable biomarkers and evidence-based treatments. Due to these difficulties, the exact cause of CRPS is still not fully understood, making it difficult to develop effective, specific treatments and conduct targeted research.

## 1. Introduction

Complex regional pain syndrome (CRPS) is an idiopathic, highly debilitating chronic disorder with persistent regional pain accompanied by a combination of sensory, motor, and autonomic abnormalities [1]. It is a rare disease with an incidence rate of 26.2 per 100,000 person-years. CRPS usually affects the extremities, primarily the distal limbs. Women are more than three times more likely to develop CRPS, with the highest incidence occurring between 61 and 70 years of age [2]. However, precipitating events (e.g., wrist fractures, carpal tunnel syndrome) may occur more frequently in some other age or gender groups.

Researchers are working to establish a set of universal outcome measures for CRPS studies [3]. The Budapest criteria, endorsed by the International Association for the Study of Pain (IASP), remain the gold standard for diagnosis, with sensitivity ranging from 45% to 99%, depending on clinician expertise [4].This variability highlights the need for objective diagnostic tools to complement clinical assessment [5].

Much of the pathophysiology and molecular processes underlying CRPS have not yet been fully elucidated. In terms of responses to acute trauma/stress, the inflammatory response involving various cytokines and autoantibodies plays a significant role. The pathophysiology of the chronic phase is more complex, involving the central and peripheral nervous system and immunological and vascular mechanisms. Preclinical data suggest an active involvement of the immune system, especially anti-inflammatory processes, in pain relief and healing in CRPS [6,7,8]. However, the results of studies on the molecular mechanisms are still controversial. Predictors of pain severity and/or relief in CRPS are urgently needed for early treatment adjustment, whether clinical or molecular [9].

Since there are still no specific laboratory diagnostic tools for CRPS, it is therefore mainly diagnosed clinically.

CRPS research is hampered by variability in the use of diagnostic criteria, a lack of standardized outcome measures, and limited data on long-term disease progression. Identifying and addressing these issues will enable the development of more robust study designs, improve the reproducibility and reliability of findings, and facilitate the discovery of effective biomarkers and therapeutic targets. Ultimately, such efforts will lead to improved diagnostic accuracy, improved patient care, and more targeted treatment strategies [9,10].

The primary objective of this scoping review is to examine recently published articles on CRPS for challenges that investigators have encountered in conducting their studies. By conducting such a review, we hope to establish a roadmap to address the most pressing issues in CRPS diagnostics. The aim of the review was to analyze the literature and summarize published data on the latest advances in clinical and molecular diagnostics of CRPS, looking for challenges that researchers have encountered in conducting research.

## 2. Materials and Methods

### 2.1. Searching Strategy

**Figure 1:**
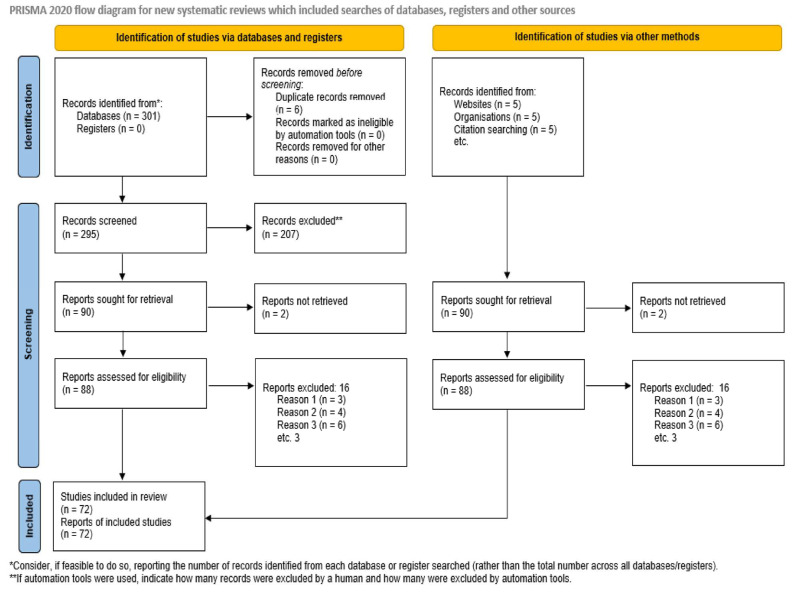
PRISMA 2020 flow diagram. Source: [13]. This work is licensed under CC BY 4.0. To view a copy of this license, visit https://creativecommons.org/licenses/by/4.0/ (accessed on 3 March 2025).

The scoping review protocol was developed according to the literature [11] and PRISMA-ScR [12] (Figure 1).

The following databases were searched: PubMed, EBSCO, CINAHL, Cochrane Library, and SCOPUS. Three independent reviewers (Z.A., J.W., and I.A.) screened, extracted data, and independently assessed the risk of bias in included studies. The JBI critical appraisal checklist was used. In case of disagreement between reviewers, a supervising associate professor decided on the inclusion or exclusion of an article.

Combinations of the following search keywords and MeSH terms were used: “complex regional pain syndrome”, “CRPS”, “algodystrophy”, “reflex sympathetic dystrophy”, “Sudeck” AND “clinical”, “molecular”, “neuroinflammation”, “cytokines”, “inflammation mediators”, “microRNA”, “biomarkers” AND “challenge” OR “limitation”.

Our search strategy focused mainly on obstacles encountered by other clinical researchers in CRPS diagnosis. We decided to include only recent papers and those that are either clinical in nature or focus on the topic of challenges and limitations of CRPS research.

We included publications meeting all of the following criteria:

1. Clinical studies with CRPS as the main research interest (mentioned in the name of the paper);
2. Including at least three patients with a diagnosis of CRPS;
3. Describing challenges the research team encountered;
4. Published after January of 2018 in English.

The search strategy was executed on the 15 April 2024, with several new eligible papers added to the review.

Studies were excluded if they had not been conducted in a clinical setting, were conducted on CRPS models, or were determined to be of very poor quality by at least two reviewers. Articles were also excluded from the review if only the abstract was available, if they were not focused on or relevant to CRPS, or if they were sociological studies rather than clinical research. Such criteria allowed us to focus on the clinical aspect of CRPS research and discard the secondary literature. The time criterion was established to search only for recent and relevant issues.

### 2.2. Data Charting Process

We charted data from research papers with the use of a form registered with the protocol. The data had been charted independently by two reviewers, then cross-checked. A unified version was agreed upon in consultation with the associate professor.

Data items.

The sources were analyzed to obtain the following variables:

1. Author and date: all listed authors and year of publication.
2. Title of the study.
3. Study type: available information on the study design to confirm the clinical setting and original nature of the study.
4. Number of subjects: only patients with CRPS were included in our review; we did not include healthy controls or patients with other diagnoses.
5. Diagnostic tools used: all tools and measurements utilized by authors to diagnose CRPS and assess disease activity, inflammation mediators, biomarkers, signs, and symptoms; we excluded measurements not pertinent to CRPS, performed solely for the purpose of the study.
6. Challenges encountered: both expected and unexpected issues that were deemed by authors to be important enough to be included in the research paper; these include direct drawbacks in conducting CRPS research, methodological shortcomings, bias, confounding factors, insufficient information, etc. We included the authors’ opinions but excluded referenced statements.

### 2.3. Synthesis of Results

Data gathered by charting was later analyzed to best answer our research questions. A spreadsheet was devised to incorporate the charted data. The number of patients was analyzed for maximum, minimum, mean, and median values. The study type was assessed to determine the percentage of interventional studies.

To summarize the challenges research encountered in CRPS and described by authors, we developed a list of broader issues for clinical and for molecular studies, based on the initial findings of our data charting process and our own clinical experience. We aimed to best encompass all possible challenges in a way that the categories do not overlap and encompass all challenges encountered in the literature. We counted the number of papers describing challenges within each category as a percentage of reviewed articles and developed a descriptive semi-quantitative analysis. Conclusions drawn are based on discussing the results among reviewers and confronting them with the associate professor.

## 3. Results

**Figure 2:**
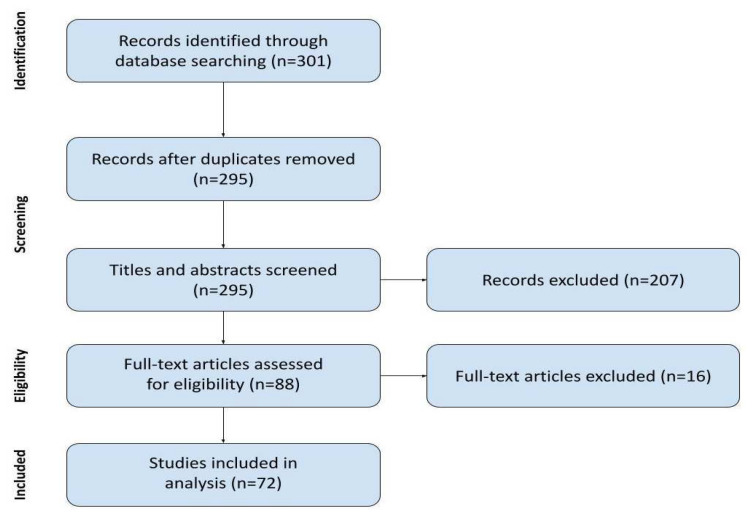
Flow diagram of the literature review process.

With the initial search, we identified 301 records. Six duplicates were removed. During the screening of titles and abstracts, we excluded 207 records of clinical studies that did not meet our inclusion criteria for apparent reasons, such as being secondary research or not focusing on CRPS. From the eighty-eight records subjected to full-text analysis, sixteen were excluded: two were only a study protocol, one was a study on only the social aspects of CRPS without any research challenges described, three were excluding CRPS patients, four were not accessible beyond the abstract by any means available to us, and six omitted the researcher challenges described. Finally, 72 studies were included in the current review. The process is presented in Figure 2.

### 3.1. Study Characteristics

**Figure 3:**
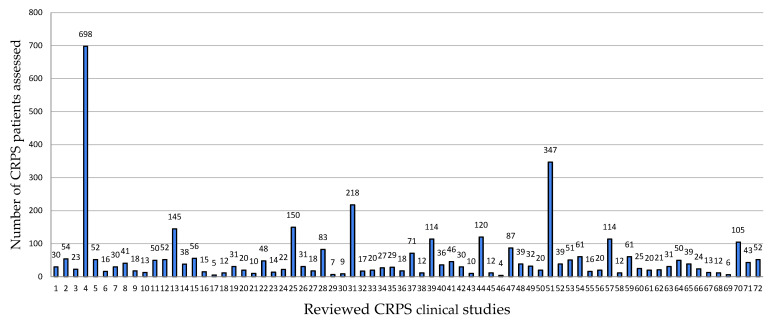
Number of CRPS patients included.

Within the investigated period (January 2018–April 2024), we found 55 original clinical research papers on CRPS and 17 studies on the immunological/biochemical/molecular aspects of CRPS. The number of CRPS patients included in studies (complete datasets) ranged from 4 to 698, with a mean of 59.40 and a median of 30 (as seen in Figure 3). The results are significantly influenced by a large cross-sectional study with 698 patients. Interventional studies constitute 53.44% of the records [14].

### 3.2. Diagnostic Tools Used

We grouped the clinical tools into three categories: patient-reported outcome measures (self-reported questionnaires), clinician-reported outcome measures (requiring trained clinicians but not equipment), and clinical measures (requiring equipment).

Among the methodologies employed in immunological/biochemical/molecular research were the following groups: cytokines/inflammatory markers, miRNA, and biomarkers of therapy effectiveness. Methodologies used in the different studies included the following: skin biopsy (utilized in two studies), serum analysis for bone health markers such as alkaline phosphatase (AP), 25-hydroxyvitamin D (25-OH vitamin D), osteoprotegerin (OPG), and markers of type I collagen turnover. Cytokine concentrations were quantified using multiplex cytokine assay panels (Life Technologies, Carlsbad, CA, USA) in conjunction with Luminex technology, immunoassays (Quantikine HS ELISA HSTA00E, R&D Systems, Minneapolis, MN, USA), and/or PeliKine compact human enzyme-linked immunosorbent assay (ELISA) kits (Sanquin Reagents, Amsterdam, The Netherlands). Oxidative and antioxidative statuses were assessed through the Erel method, which evaluates total oxidant status (TOS) and total antioxidant status (TAS).

### 3.3. Patient-Reported Outcome Measures

In CRPS studies, self-reported questionnaires were the most common assessment tool, with 59 different methods reported. However, only 23 of these appeared in more than one study.

Pain Assessment: Pain self-assessment was very common (74.5% of studies), with ten different methods used. The most frequent were the Numeric Rating Scale (NRS) and Visual Analog Scale (VAS) [14,15,16,17,18,19,20,21,22,23,24,25,26,27,28,29,30,31,32,33,34,35,36,37,38,39,40,41,42,43,44,45,46,47,48,49,50,51,52,53,54]. Other methods included the Brief Pain Inventory (BPI) [17,55], McGill Pain Questionnaire (MPQ) [14], and PainDetect Questionnaire for neuropathic pain (PDQ) [17,55,56,57,58,59,60].

Pain’s Impact: The effect of pain and pain coping were assessed using eight different methods across nine studies. The Pain Catastrophizing Scale (PCS), Pain Anxiety Symptoms Scale (PASS-20), and Pain Disability Index were used [14,17,18,20,21,28,37,44,55,56,57,61].

Psychological Assessments: Thirteen different psychological assessments were used across 17 studies. Common ones included the Tampa Scale for Kinesiophobia, Hospital Anxiety and Depression Scale (HADS), and Bath CRPS Body Perception Disturbance Scale (B-CRPS-BPDS). Others assessed depression, anxiety, and stress [14,17,18,19,20,21,28,37,39,44,55,56,57,61,62,63,64].

Functional Questionnaires: Twelve functional questionnaires measuring activities of daily living were used in nine papers. Disability of the Arm, Shoulder, and Hand (DASH) was the most common, despite only applying to upper limbs [55,56,61,65,66,67].

Quality of Life: Eleven different quality of life questionnaires were used across 24 papers. The Patient Global Impression of Change and the SF-36 Health Survey were the most common [23,29,55,56,61,65,66,67].

**Figure 4:**
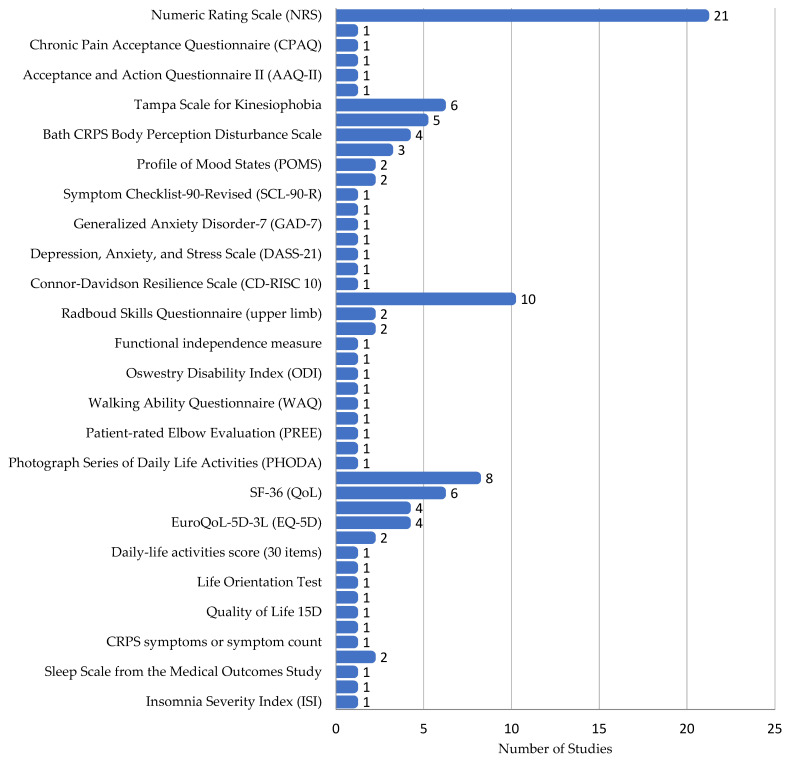
Patient-reported outcome measures.

Several other questionnaires were used only once each, including those assessing CRPS symptoms, handedness, sleep, insomnia, and cold intolerance (Figure 4).

### 3.4. Clinician-Reported Outcome Measures

In CRPS studies, clinician-reported outcome measures involved 14 different methods, primarily focused on diagnosing CRPS (48 studies) [14,15,16,17,18,19,20,21,22,23,24,25,26,27,28,29,30,31,32,33,34,35,36,37,38,39,41,42,43,44,45,46,47,48,49,50,51,52,53,54,55,56,57,58,59,60,61,62,63,64,65,66,67,68,69,70,71,72,73]. The Budapest criteria were most frequently used (44 studies).

**Figure 5:**
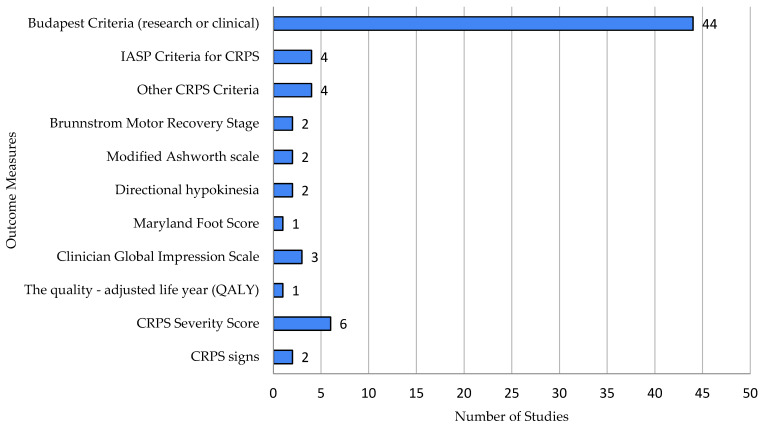
Clinician-reported outcome measures.

Motor Function: Four assessments were used across five studies, including the Brunnstrom Motor Recovery Stage and Modified Ashworth Scale.
Quality of Life: Clinician-assessed quality of life was measured in four studies using tools like the Clinician Global Impression Scale.
Neuropathic Pain: DN4 and the Mainz Pain Staging System were used.
Other CRPS Features: Three methods tracked CRPS features, including the CRPS severity score (CSS). One study assessed lateralization with a Mental Number Line Bisection task (Figure 5).

### 3.5. Clinical Measures

**Figure 6:**
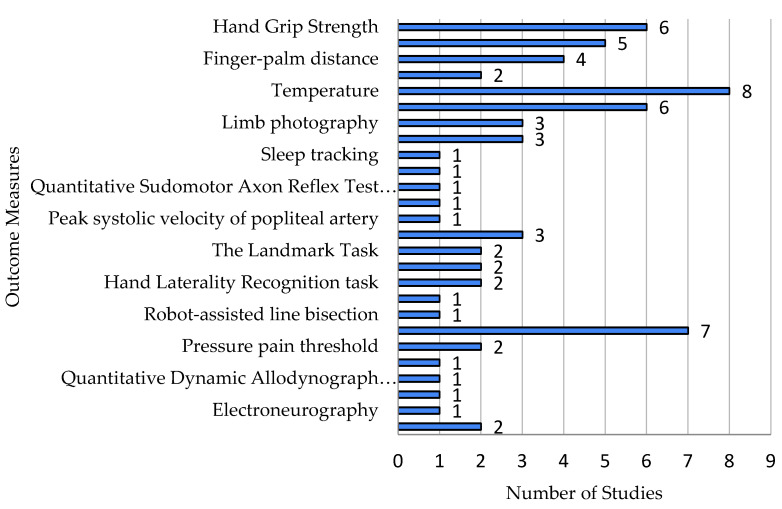
Clinical measures.

Clinical measures requiring equipment included 26 different methods (Figure 6).

Motor function: Four methods were used across twelve papers, including hand grip strength (dynamometer), range of motion (goniometer), finger–palm distance (measuring tape), and wrist accelerometry (wearable device).

Sympathetic abnormalities: Nine measures were used across fifteen studies. The most common were temperature (infrared thermometer) and edema (measuring tape or volumetry). Other methods included limb photography, bone scintigraphy, sleep tracking, heart rate response, QSART, head-up tilt, and popliteal artery velocity.

Cognitive function: Six methods were used across six papers, primarily computer-based tasks and lateralization assessments.

Sensory function: Four methods were used across eleven papers, including quantitative sensory testing (von Frey filaments, pinprick, and allodynia), the pressure pain threshold (algometer), the electrical sensory threshold, and the quantitative dynamic allodynograph.

Electrophysiological measures: Two studies used EEG and ENG.

Impairment Level Sum Score (ISS): Two studies used this complex assessment, which includes pain, temperature, volume, and range of motion.

### 3.6. Immunological, Biochemical and Molecular Aspects of CRPS

The exact sequence of events leading to the development of CRPS remains unclear. Nevertheless, given their critical role in normal trauma response and chronic pain [74,75], pro-inflammatory cytokines such as necrosis factor-alpha (TNF-α), interleukin (IL)-6, and IL-1 are believed to play a significant role in the pathophysiology of CRPS. Numerous studies highlight the crucial role of neuropeptides in the pathophysiology of CRPS. In human “warm” CRPS, the electrical stimulation of primary afferents induces pronounced neurogenic flare and significant edema. The flare is thought to be mediated by an endogenous calcitonin gene-related peptide (CGRP), while the edema is attributed to endogenous substance P (SP) [76]. Moreover, the detection of autoantibodies targeting an inducible antigen of the autonomic nervous system suggests that CRPS may have an autoimmune component [77].

Most studies evaluating the role of pathophysiological processes in the development and progression of CRPS were conducted in experimental models. Clinical studies of molecular changes in patient groups are quite scarce. According to our criteria, 17 articles were included in the review. Among them, three were clinical studies, two were observational studies, three were cross-sectional studies, one was a retrospective study, seven were experimental studies, and one was a case–control study. Methodologies used in the different studies included skin biopsy (used in two studies), serum analysis to determine markers of bone health such as alkaline phosphatase (AP), 25-hydroxyvitamin D (25-OH vitamin D), osteoprotegerin (OPG), and markers of type I collagen turnover. Cytokine levels were quantified using multiplex cytokine assay panels (Life Technologies, Carlsbad, CA, USA) coupled with Luminex technology, immunoassays (Quantikine HS ELISA HSTA00E, R&D Systems), and PeliKine compact human enzyme-linked immunosorbent assay (ELISA) kits. Oxidative and antioxidant status were assessed using the Erel method, which assesses total oxidative status (TOS) and total antioxidant status (TAS), a dabsyl-bradykinin (DBK)-based assay. All studies required trained personnel and additional equipment to perform. The main limitations encountered by the investigators were mostly related to limitations of the laboratory methodologies and inherent limitations of the study design.

#### 3.6.1. Cytokines/Inflammation in CRPS

CRPS typically develops following an injury that triggers a localized inflammatory response in skin cells and resident immune cells within the affected area. This local inflammatory reaction can upregulate α1-adrenergic receptor (α1-AR) expression in skin cells and nerve fibers of the affected limb [78,79,80,81]. In response to injury, circulating immune cells such as peripheral blood mononuclear cells (PBMCs) are recruited to the injury site, initiating complex inflammatory cascades that normally facilitate wound healing. However, in CRPS patients, this process may amplify inflammatory responses, contributing to persistent localized and regional inflammation. It has been documented that the initial 12 to 18 months of CRPS are characterized by the production of inflammatory cytokines within the affected limb [82,83,84,85,86]. This persistent inflammatory state may sensitize nociceptors and exacerbate pain, partially mediated by α1-ARs expressed on nerve fibers and other cell types surrounding the site of injury [78,79,80]. Postganglionic sympathetic neurons innervating immune organs, such as the thymus and spleen, regulate immune cell activity via adrenergic receptors [87]. Specifically, α1-AR signaling modulates cell migration and the production of inflammatory cytokines [88,89,90,91,92]. In PBMCs from healthy individuals, α1-AR expression is low or undetectable [92,93,94,95,96]; however, in chronic inflammatory diseases such as juvenile rheumatoid arthritis, PBMCs express α1-ARs, and stimulation with the α1-AR agonist phenylephrine enhances the production of the inflammatory cytokine interleukin-6 (IL-6) [90,92]. IL-6 promotes the differentiation of B lymphocytes into plasmablasts, thereby increasing antibody production [38,39]. This process may ultimately generate autoantibodies, contributing to autoimmune responses in chronic inflammatory diseases [97,98,99,100,101].

In a subset of patients with CRPS, an overexpression of α1-ARs has been observed. The activation of these receptors in epidermal cells enhances the production of the pro-inflammatory cytokine IL-6. A recent study aimed to assess whether these interactions contribute to the exacerbation of inflammation in CRPS [81]. Primary keratinocytes or fibroblasts were isolated from skin biopsies of patients and healthy controls. To induce an inflammatory state, the cells were treated with tumor necrosis factor-alpha (TNF-α) and subsequently incubated with the α1-AR agonist phenylephrine. Exposure to TNF-α stimulated the production of pro-inflammatory cytokine mRNA and protein secretion in keratinocytes and fibroblasts while also increasing the expression of α1B-AR mRNA in keratinocytes. Further stimulation of α1-ARs with phenylephrine augmented IL-6 mRNA production and protein secretion in both cell types. Across all conditions, the levels of α1-AR mRNA and protein, as well as cytokine gene expression and protein secretion, were generally comparable between patients and controls. However, three of the seventeen patients exhibited abnormally high α1-AR protein levels in keratinocytes.

These findings suggest that persistent inflammation in CRPS is not due to the intrinsic dysfunction of skin cells but represents a normal cellular response to external stimuli. Notably, IL-6 mRNA elevations, but not protein levels, in keratinocytes following α1-AR stimulation were proportional to baseline α1-AR protein levels. This implies that skin cells play a significant role in chronic inflammation associated with CRPS. A potential positive feedback loop between α1-ARs and IL-6 production in skin cells may contribute to the maintenance of this inflammatory state.

In another study, Wijaya assessed the effect of α1-AR stimulation pro-inflammatory cytokine production by PBMCs [102]. Lipopolysaccharide (LPS), a bacterial toxin, was administered to cultured peripheral blood mononuclear cells (PBMCs) for 24 h to induce inflammation. It has been shown that the interplay between the increased expression of α1-ARs in PBMC and IL-6 secretion may contribute to systemic inflammation and increase IgG production in differentiated B lymphocytes (plasmablasts) that bind to α1A-AR, β2-AR, and muscarinic receptors-2 in patients with CRPS. This suggests a potential link between inflammation and adrenergic receptor activity in CRPS, which may contribute to a better understanding of the mechanisms of the disease. The study further demonstrated that the stimulation of α1-adrenergic receptors (α1-ARs) with phenylephrine during LPS-induced inflammation selectively enhanced IL-6 production in PBMCs, without affecting IL-1β levels. This adrenergic influence appears to be specific to IL-6. These findings align with earlier research in keratinocytes, where a feedback mechanism between inflammatory cytokines and α1-AR expression was shown to amplify the production of the pro-inflammatory cytokine IL-6 [81,103].

The authors speculated that the upregulation of α1-AR expression may increase susceptibility to CRPS. Specifically, they suggested that elevated α1-AR expression in PBMCs and other cells at the injury site could heighten their sensitivity to α1-AR ligands, enhancing IL-6 production and sustaining inflammation. Adrenergic-induced IL-6 production in PBMCs may also contribute to systemic disturbances in CRPS. Previous studies have reported the bilateral upregulation of α1-AR expression in the epidermis and cutaneous nerve bundles of CRPS type I patients compared to pain-free controls [80]. Similarly, increased α1-AR expression was observed bilaterally in the plantar epidermis, skin blood vessels, and sciatic nerves in a tibial fracture rat model of CRPS type I [78]. Notably, systemic injection of the α1-AR antagonist prazosin reduced pain in these animals, whereas localized injections had no effect.

Another key finding of this study was that IL-6 administration increased IgG production in both untreated and stimulated PBMCs in vitro.

CRPS patient serum contains autoantibodies targeting α1A-AR, β2-AR, and muscarinic-2 receptors [104,105], and the passive transfer of this serum to mice replicates CRPS-like symptoms [106,107,108,109,110]. Conversely, intravenous immunoglobulin therapy, commonly used to normalize immune function in autoimmune conditions, alleviates pain in some CRPS patients [111,112,113]. Although the mechanisms underlying autoantibody production in CRPS remain unclear, studies in a tibial fracture mouse model suggest that B cell activity, generating antibodies against neoantigens, contributes to CRPS-like pain behaviors [114]. IL-6 signaling after injury plays a role in nociceptive sensitization and lymph node activation, characterized by germinal center responses and local IgM production [115].

Further research into the interaction between α1-AR, IL-6, and IgG production in PBMCs, using complex systems such as animal models or clinical studies, could clarify whether CRPS PBMCs exhibit heightened or distinct antibody responses to pro-inflammatory cytokines like IL-6 in vivo.

Parkitny et al. assessed the influence of the immune response on the later development of CRPS [116]. Depression, anxiety, and stress were assessed using the 21-item Depression, Anxiety, and Stress Scale (DASS-21). Cytokine levels were measured using 25-plex human cytokine panels (Life Technologies, Carlsbad, CA, USA) and Luminex technology. Unfortunately, no evidence indicating that the early expression of systemic cytokines after injury may be associated with the diagnosis of CRPS 16 weeks after injury was found. These findings indicate that post-fracture serum cytokine levels were not associated with a later diagnosis of CRPS. This challenges the hypothesis that the acute systemic immune response to fracture plays a direct role in the development of CRPS. The authors noted one limitation of the study as the testing of systemic cytokines, noting that the use of complementary multi-omics approaches would provide additional insights.

Indirect confirmations of the fact that inflammatory processes are important in the pathogenesis of CRPS include the observations of Kalita J. et al., who showed that treatment with prednisolone at a dose of 20 mg and 40 mg contributed to a reduction in pain intensity (measured using the Visual Analog Scale (VAS) and a reduction in the severity of CRPS [117].

Karpin H. et al. [118] presented a cluster analysis of the psychological and biological (pro-inflammatory) profiles of patients with CRPS. It was shown that pain hypersensitivity and psychological measures can be performed to distinguish CRPS from other chronic limb pain (CLP) conditions, and the pro-inflammatory cytokine tumor necrosis factor-alpha (TNF-α) is an additional potential biomarker as the serum level of TNF-α was higher in the ‘CRPS’ vs. ‘CLP’. The limitations of this study were related to the pain measurement techniques, in particular to the lack of a contingent pain modulation test and contralateral limb pain assessment.

#### 3.6.2. miRNA

Preclinical data suggest an active involvement of the immune system, particularly anti-inflammatory processes [119,120,121]. These include T-cell and cytokine regulation as well as neuroimmune interactions. Identifying predictive factors for pain relief in CRPS is urgently needed to tailor treatments at an early stage, whether clinical or molecular.

One potential molecular predictor is microRNA (miRNA), which has been shown to act as a key regulator in the development and maintenance of various pain syndromes, often targeting the immune system [72].

Exosomal miRNAs seem particularly valuable in this context as potential biomarkers due to their stability and dysregulation in diseases including CRPS, a chronic pain disorder with persistent inflammation.

Numerous studies have analyzed miRNA profiles in patients with CRPS.

Orlova I.A. et al. identified three distinct groups of whole-blood miRNA profiles in forty-one CRPS patients, with eighteen miRNAs showing differential regulation. Interestingly, these miRNAs were also present in serum exosomes. However, in a smaller cohort of 6 CRPS patients and 6 healthy controls, these miRNAs were not significantly regulated, unlike 127 other differentially expressed miRNAs. Among these, hsa-miR-939-5p showed increased expression, reducing pro-inflammatory mediators like IL-6 and nitric oxide synthase 2 in vitro [122].

In the study by Dietz, Ch. et al. [123] clinical phenotypes, sensory profiles, patient-reported outcomes, and exosomal immune barrier microRNAs (miRs) regulating barrier function and immune response were compared between CRPS and fracture controls. Dietz’s study highlighted alterations in miRNA expression in CRPS, including hsa-miR-223-5p and hsa-miR-144-5p, which regulate barrier integrity and inflammation. Reduced levels of miR-223-5p, known for its anti-inflammatory and neuroprotective properties, have been identified in blood exosomes of CRPS patients compared to those who did not develop CRPS following trauma (“fracture controls”). miR-223 was downregulated in CRPS and negatively correlated with CRPS pain and severity. Additionally, miR-223-5p showed a negative correlation with both edema and the CRPS severity score (CSS). Another anti-inflammatory miRNA found to be reduced in CRPS is miR-939 [124].

MiR-223, a highly conserved miRNA in myeloid lineage cells, supports granulocyte function, negatively regulates neutrophil activity and chemotaxis, promotes M2 macrophage polarization, and enhances Schwann cell proliferation during regeneration [125]. In normal healing, hsa-miR-223-5p was upregulated, negatively correlated with the CRPS severity score (CSS), and reduced in patients with edema. Similarly, hsa-miR-223-3p, the leading strand of the miRNA duplex, has been linked to a lower risk of chronic pain after lumbar disk herniation and attenuates neuronal activity in pain pathways in animal models. Studies suggest that the leading (−3p) and passenger (−5p) strands of miR-223 are co-regulated.

Moreover, hsa-miR-223 has been implicated in autophagy [126] and shows a negative correlation with macrophage and microglial activity [127], which is relevant in CRPS pathophysiology, particularly regarding osteoclast-driven bone loss or osteoporosis, a hallmark of the condition. On the other hand, miR-144, primarily studied in cancer and viral diseases [128,129], demonstrates varied effects depending on the tissue or condition. While it can reduce oxidative stress and promote apoptosis in neurons under oxygen and glucose deprivation, it also protects against pulmonary vascular damage and intestinal barrier disruption. However, no significant role for miR-144 in pain conditions has been observed, consistent with the findings of this study.

So, it may be concluded that hsa-miR-223-5p, but not hsa-miR-144-5p, emerges as a potential biomarker with protective effects against CRPS and edema formation. Although the exact mechanisms by which miR-223-5p might influence barrier dysfunction remain unclear, previous research by Albrecht et al. [130] demonstrated disrupted endothelial integrity in arterioles and capillaries of amputated limbs with CRPS. CGRP, a neuropeptide, may play a role in barrier disruption in CRPS. Computational target scans suggest a potential link between miR-223-5p and CGRP via the CALCB gene, indicating that reduced miR-223-5p expression in CRPS patients could lead to elevated CGRP levels, contributing to edema through barrier dysfunction. However, these hypotheses require further validation through in vitro and in vivo studies.

Whether the analysis of selected miRNAs such as hsa-miR-223-5p will help in further differentiation is likely but needs to be confirmed in future longitudinal studies. The authors mentioned that miR analysis is very resource-intensive, and this was one of the limitations of the study.

#### 3.6.3. Biomarkers of Therapy Effectiveness

Various studies have attempted to identify biomarkers that could be useful in predicting the course of the disease/response to treatment.

In the study by Ann Kristin Reinhold et al. [72], patients were included within 2.5 years of receiving standard treatment. The study focused on the prognostic significance of miR-223 expression in predicting pain relief. At the same time, the Neuropathic Pain Symptom Inventory (NPSI), Quantitative Sensory Test (QST), and Beck Depression Inventory II (BDI II) were assessed. The authors identified a gradual decrease in miR-223 during treatment, suggesting that miR223 could be considered as a potential biomarker of chronic CRPS pain and changes in pain intensity during treatment. Interestingly, miR-223 levels displayed a contrasting pattern: the relief group exhibited an upward trend in miR-223 levels. This suggests that pain relief is not a passive occurrence but rather an active process. The observed negative correlation between miR-223 dynamics and pain progression over time further supports the protective role of miR-223 in CRPS-related pain. Clinically, this study highlights the importance of early diagnosis. The possibility of pain relief was associated with a shorter disease duration and correlated positively with severe initial pain in this study, but the authors emphasize the need for the standardization of treatment and follow-up procedures.

Ramanathan S. et al. [76] investigated exosomal miRNAs as a strategy for patient stratification to maximize therapeutic outcomes. Plasma cytokine levels were measured by HPLC and correlated with miRNA expression. A Luciferase assay was used to assess the miRNA-mediated regulation of the target mRNA gene after the co-transfection of HEK293 cells with targeted 3′UTR constructs and miRNA mimics. The study indicated that the analysis of exosomal miRNA before and after therapeutic plasma exchange (PE) may be useful for identifying molecular markers important for predicting treatment response. Lower levels of miR-338-5p before treatment in poor responders were associated with IL-6 levels and inflammation in CRPS. In vitro validation studies confirmed an interaction between miR-338-5p and IL-6. Plasma IL-6 levels were higher in patients who responded to treatment before PE and significantly decreased after PE. This suggests that the removal of IL-6 through PE may directly contribute to reducing inflammation. The findings of this study, which differentiate responders from poor responders, indicate that higher pre-PE levels of IL-6 in responders are associated with miR-338-5p levels. In other words, elevated IL-6 levels in responders may mediate intercellular signaling via exosomal miR-338-5p to resolve inflammation. When pro-inflammatory IL-6 is removed from circulation during PE, exosomal miR-338-5p levels also significantly decrease. Conversely, poor responders showed no significant reduction in plasma IL-6 levels post-PE, resulting in no substantial changes in the exosomal miR-338-5p levels. In summary, these findings suggest that measuring miRNA expression levels in exosomes alongside cytokine analysis could be a viable approach for patient stratification and may aid in predicting the therapeutic efficacy of PE. A limitation of this study was the lack of analysis across a broad range of immune markers, which would be necessary to further evaluate and extend these initial observations.

Biomarkers were sought in three clinical trials to assess the efficacy of pharmacological therapy.

The study by Dinç M. et al. [131] aimed to assess the efficacy of N-acetylcysteine in preventing CRPS-1 by reducing pro-inflammatory cytokines and oxidative stress markers in the serum of patients with distal radius fractures. This study highlights the potential of N-acetylcysteine as a preventive treatment for CRPS-1 and emphasizes the importance of early intervention.

Kalita J. et al. [117] assessed the role of corticosteroids in CRPS and their effect on serum cytokine levels in patients. Prednisolone 20 mg was effective in patients with CRPS, was non-inferior to 40 mg in CRPS-I, and was safe in patients with diabetes. The main limitation of these studies was the lack of standardization of the therapeutic approaches. The microcirculation status and biomarkers of epithelial and endothelial function were also significant.

Skin biopsies offer a window to analyze the somatosensory and vascular systems, as well as skin trophicity with its protective barriers. Skin biopsies were used in two studies. In the study by Mehling K. et al. [132], microvessels, somatosensory receptors, barrier proteins, including claudin-1, claudin-5, and claudin-19, as well as Meissner bodies, Merkel cells, and intraepidermal nerve fiber density were assessed in skin biopsies. This study showed the bilateral loss of papillary vessels and Meissner bodies in acute CRPS skin without differences in barrier proteins (claudin-1, claudin-5, and claudin-19).

König S. et al. [133] developed a dabsyl-bradykinin (DBK)-based assay and used it to study CRPS patients. The main result is that the degradation of DBK to fragments 1–8 and 1–5 in the healthy control and dPNP is shifted to higher values for DBK1–8 and lower values for DBK1–5 after 1 h of incubation in CRPS patients.

Autoantibodies directed against human β2-adrenergic and muscarinic M2 receptors (hβ2AR and hM2R) have been previously described in CRPS patients, further supporting the hypothesis of immunological pathophysiology. Dharmalingam B. et al. [75] analyzed the sera of CRPS patients for autoantibodies against hβ2AR, hM2R, and endothelial cells and investigated the effect of purified IgG from 13 CRPS patients on endothelial cells. They showed that not only do patients with CRPS develop autoantibodies to hβ2AR and hM2R, but these antibodies also disrupt endothelial cell function in vitro and thus may contribute to the pathophysiology of CRPS.

Bone loss is also considered a characteristic clinical feature of CRPS. The altered bone microstructure in patients with CRPS is thought to be due to increased bone resorption via increased osteoclast differentiation and activation. Bone metabolism was assessed in a study by Harnik M.A. [134] using measurements of alkaline phosphatase, 25-OH vitamin D, osteoprotegerin, procollagen type I N-terminal propeptide, and β-C-terminal telopeptide. The authors concluded that most serum markers remained unchanged, but patients with warm CRPS showed unique features, suggesting distinct pathophysiological profiles. The main limitations of these studies were related to the skin biopsy technique, the heterogeneity of the obtained material, and insufficient conditions for the assessment of associated factors.

## 4. Challenges in Clinical and Immunological/Biochemical/Molecular Studies

### 4.1. Challenges Encountered by Authors in Clinical Studies

To summarize challenges in CRPS research encountered and described by authors, we developed a list of 16 broader issues, based on the initial findings of our data charting process and our own clinical experience. We aimed to best encompass all possible challenges so that the categories do not overlap and do encompass all challenges encountered in the literature. We list here, in decreasing recurrence order, the challenges, with a short commentary on recurrence, criteria, and findings:

1. Difficulty in recruiting participants (60% of the studies)

This common category included all cases of the reported unsatisfactory number of study subjects, be it by design, inability to recruit, or drop-out. CRPS is a relatively rare condition, making it difficult to recruit a large enough sample for clinical trials so they are often designed for a small number of participants (as shown previously in the section “Number of patients and study type”).

2. Difficulty in controlling for confounds (60% of the studies)

There are a lot of confounding factors to control when conducting CRPS research. These include co-occurring medical conditions and medications, placebo effects, psychological factors, such as stress, anxiety, or level of cognitive functioning, and lifestyle factors, such as diet and physical activity. The lack of data on the subjective weight of the different confounds makes controlling the factors more difficult. Combined with the small number of participants, this results being from a highly heterogeneous study population makes assessments of the interventions’ efficacy extremely difficult. This category does not include issues arising due to the heterogeneity of CRPS features.

3. Heterogeneity and complexity of the disorder (49% of the studies)

CRPS can present differently in different individuals and its features change in time, making it challenging to design and conduct studies that fully capture the full spectrum of the disorder and ensure adequate generalizability of the results. There are known subtypes of the disease (CRPS-I and CRPS-II), with no clear indication of whether they should be dealt with similarly or differently. This category does not include other disorders that may co-occur with CRPS, such as anxiety or depression.

4. Difficulty in measuring outcomes (47% of the studies)

Several features of CRPS, especially pain, are subjective and can be challenging to quantify, which makes it difficult to accurately measure the effects of interventions in CRPS research. This is further complicated by commonly missing data items, due to pain interrupting the assessment. This category does not include issues relating to insufficient assessment methods or the insufficient reporting of autonomic symptoms, focusing only on difficulties in using existing assessment tools.

5. Poor quality of existing data (44% of the studies)

The quality of existing data on CRPS is often poor, making it challenging to conduct systematic reviews or meta-analyses to summarize the current state of knowledge. Information on intervention efficacy usually comes from small, limited studies or expert opinions, which makes it difficult to compare the effectiveness of different interventions. Patients’ medical records are highly heterogeneous due to a lack of reporting standards for CRPS, are rarely reported autonomic abnormalities, and are commonly incomplete, which makes it challenging to perform retrospective analyses. This category does not omit data due to interrupted assessments or patients dropping out.

6. Lack of standardized, validated assessment tools (40% of the studies)

There is no definitive test for diagnosing CRPS, and the diagnosis has to be made based on clinical examination, which can make it challenging to ensure consistency and accuracy in diagnosis across studies. There is also a lack of agreement on the best way to measure outcomes in CRPS research and a limited availability of validated patient-reported outcome measures for CRPS, which makes it challenging to compare results across studies. This category relates to the absence of proper tools to diagnose and assess disease activity, not to the technical difficulties of the available methods.

7. Difficulty in conducting long-term treatment and follow-up (27% of the studies)

CRPS is a chronic condition that can persist for many years, making it challenging to conduct long-term follow-up studies to evaluate the durability of treatment effects. Many of the investigated studies pointed out the observation period being too short and the need for a longer follow-up of 2–3 years. This category does not include cases of patients dropping out of the study.

8. Variability and lack of standardization in treatment approaches (25% of the studies)

There is a lack of standardization in the treatment of CRPS, and treatment approaches vary on both national and international levels, which results in patients receiving different therapies depending on where they are treated. This makes it challenging to design widely generalizable high-quality studies. This category applies only to heterogeneity in treatment options and not to diagnostic methods.

9. Difficulty in blinding the study (11% of the studies)

Treatment options for CRPS include non-pharmacological interventions like physical therapy, psychotherapy, and invasive procedures like nerve blocks, which are difficult to blind and may have a significant placebo effect. This results in a non-controlled, non-blinded design of several studies for promising, non-pharmacological interventions. This category does not include difficulties with controlling for placebo effects.

10. Ethical considerations (7% of the studies)

Conducting clinical trials in individuals with CRPS can pose ethical challenges, particularly when the interventions being tested have the potential to cause harm or when providing sham interventions to patients in severe pain. Due to the uncertain efficacy of used interventions, the risk–reward ratio should be evaluated for each patient individually, which makes designing high-quality interventional studies difficult. Patients with severe pain, especially with intractable CRPS, may be more eager to undertake a higher-than-minimal risk, and it is the researchers’ responsibility to guarantee patients’ safety.

11. Limited understanding of the underlying mechanisms (7% of the studies)

The exact cause of CRPS is still not fully understood, making it challenging to develop effective, specific treatments and conduct directional research. Most of the studies mention the unknown etiology of CRPS.

12. Inadequate training for healthcare providers (5% of the studies)

Healthcare providers may not have adequate training in recognizing and managing CRPS, which can lead to misdiagnosis and inappropriate treatment, leading to an increase in subjects’ heterogeneity and making it more challenging to conduct high-quality research. This category does not include medical records with missing data.

13. Limited access to specialized care (4% of the studies)

Access to specialized care for CRPS can be limited, particularly in rural or underserved areas, making it difficult for individuals with CRPS to participate in research studies. This highlights the challenge of participant recruitment and study result dissemination.

14. The stigma surrounding chronic pain (4% of the studies)

There can be a stigma surrounding chronic pain, including CRPS, which can lead to the underreporting of symptoms, a longer time from onset to diagnosis, and the underdiagnosis of the disease. A low social awareness of chronic pain can make it more difficult for patients to obtain adequate help and find information on ongoing clinical trials. Few studies reported this issue, possibly due to its primary social, not medical, nature.

15. Limited funding (2% of the studies)

Research into rare or poorly understood conditions like CRPS often lacks funding, making it difficult to carry out large-scale studies. Only one of the investigated studies reported insufficient financing, although we found no large-scale, high-quality interventional clinical trials within the scope of the review.

### 4.2. Challenges Encountered by Authors in Immunological/Biochemical/Molecular Studies

Studies aimed at identifying immunological/biochemical/molecular diagnostic/prognostic markers in CPRS also encounter many limitations. All studies of these testing methods required trained personnel and additional equipment to be performed, the most frequently mentioned of which are

1. Difficulty in recruiting participants (82% of the studies)

As was shown in clinical studies, CRPS is a relatively rare condition, making it difficult to recruit a large enough sample for clinical trials, so they are often designed for a small number of participants.

2. Inflammatory response assessed in vitro may not reflect the inflammatory processes in vivo (29% of the studies)

This is because in vitro models often lack the complex interactions between different cell types, tissues, and systemic factors, such as the nervous, immune, and circulatory systems, that are integral to the in vivo inflammation state. Furthermore, environmental factors, such as biomechanical forces and microbiome influences, that play a significant role in modulating inflammation in vivo are typically absent from in vitro setups. Therefore, while in vitro studies are valuable for identifying and investigating specific mechanisms, they may not fully capture the holistic, dynamic nature of in vivo inflammatory responses. Furthermore, when studying cultured cells, maintaining their physiological relevance to their endogenous state is crucial. To achieve this, cells from early passages, such as passages 2 to 3, are often used because they closely mimic the functional and structural characteristics of cells in their native environment. However, the repeated passage process can lead to significant changes in cellular behavior. For example, the production of pro-inflammatory cytokines—a key feature of inflammatory responses—has been shown to decrease after multiple passages. This reduction may impair the ability of cells to accurately reflect the in vivo conditions of certain diseases such as CRPS.

3. Findings for early CRPS might not generalize to persistent CRPS and vice versa (35% of the studies).

The findings for early CRPS do not necessarily apply to chronic CRPS and vice versa, due to distinct pathophysiological mechanisms, clinical presentations, and stages of disease progression. Early CRPS often involves acute inflammation, increased sensitivity to stimuli, and local symptoms such as redness, swelling, and warmth. In contrast, chronic CRPS may reflect a shift toward chronic pain mechanisms, including central sensitization, changes in the nervous system, and potential atrophy or dystrophic changes in the affected limb. In addition, the psychological, genetic, and systemic factors that influence CRPS may differ significantly over time, leading to differences in the symptom profiles and treatment responses between early and chronic stages. As a result, conclusions drawn from studies focused on one stage may not be directly applicable to the other, emphasizing the need for tailored research and interventions that take into account the unique characteristics of the different stages of CPRS.

4. Heterogeneity and complexity of the disorder (41,18% of the studies)

CRPS has a wide variety of signs and symptoms, making it difficult to identify pathophysiological pathways and mechanisms. CRPS is a multifaceted disorder involving systemic and localized factors, including neural, immunological, and vascular components that cannot be fully replicated in isolated cell models.

5. Difficulty in controlling for confounds (41% of the studies).

The difficulty in controlling confounding in CRPS studies is multifaceted, encompassing the interplay of biological, psychological, and social factors that may influence CRP levels and pain perception. The transient nature of CRP, the presence of multiple potential confounders, and the risk of residual confounding contribute to the challenges that investigators face in establishing clear and reliable associations in molecular studies.

6. Variability and lack of standardization in treatment approaches (47% of the studies)

Some drugs, even after discontinuation, may have residual effects on physiological processes, inflammatory markers, or neural pathways that could alter study results. For example, drugs with long half-lives or those that induce changes in receptor sensitivity, gene expression, or immune responses may continue to exert their effects well beyond the period of their active administration. In addition, the drugs may have interacted with other variables in the study, such as baseline conditions or responses to interventions, potentially confounding the results. This emphasizes the need to carefully consider treatment history and its possible long-term effects when interpreting results and designing future studies.

7. Lack of standardized, validated assessment tools (47% of the studies)

These limitations undermine the reproducibility and reliability of biomarker identification, making it difficult to draw consistent conclusions across studies. Similar barriers have been reported in clinical evaluations, where inconsistencies in methods impede the integration of laboratory findings into patient care and the development of effective, evidence-based treatments.

8. Lack of a comparison baseline, distinguishing CRPS subtypes, and experimental control (47% of the studies).

The lack of a reference point, the proper distinction of CRPS subtypes, and experimental control are key challenges in laboratory studies focusing on cytokines and inflammatory markers in patients with CRPS. These limitations undermine the validity and reliability of the results because they make it difficult to accurately interpret the data by considering subtypes or other confounding factors.

## 5. Discussion

According to the data collected, there is a strong need for new, better-quality clinical and laboratory research in the field of CRPS. In the six years surveyed, we found only 72 original clinical research papers, out of which 33 had an interventional study design. Most of the challenges researchers face are due to difficulties in obtaining high-quality empirical evidence, due to small, heterogeneous samples and inadequate assessment tools.

The main challenges in both types of studies were difficulty in recruiting participants and difficulty in controlling for confounds. Looking at the number of patients included in CRPS studies, both clinical as well as molecular, we can see that most of them are small. This is not surprising for a rare disease and was reflected in it being the most reported challenge. Planning and conducting high-quality CRPS studies are further complicated by the high internal variability of studied populations. Patients are recruited with diverse clinical features, at different stages of the disease, subject to a wide variety of confounding factors, and they are often incapable of finishing planned interventions or assessments due to severe pain. Different medical approaches could also influence the results of studies. Variability and a lack of standardization in treatment were also some of the main limitations in both types of studies. Concomitant medications, such as opioid analgesics or anticonvulsants, have been reported to influence the results to an unknown degree. Also, the duration of studies or follow-ups tends to be too short, which was the main limitation for clinical studies. So far, there is no widely adopted standard for CRPS research, although there is an ongoing effort to establish one [4,135]. A significant step forward is marked by the widespread use of the Budapest criteria [4,70] to diagnose CRPS (67 out of the 72 papers reviewed). It is worth mentioning that there are currently two versions of the criteria in use: the stricter research criteria and the laxer clinical criteria. We did not distinguish between these two in our review, as we believe that the authors chose the one best suited for their study. Moreover, the disease may present very differently in different individuals and change significantly over time. The above adds to the issue of the high heterogeneity of the study population and the limited potential for result generalization. The high internal variability of the population was repeatedly reported by the authors as it makes controlling for confounds more difficult. Common confounds included comorbidities (especially anxiety and depression), medications, and psychological and lifestyle factors. Planning research is further complicated by difficulties in blinding both patients and the investigators. A lot of investigated interventions include non-pharmacological elements, like physical therapy, or invasive procedures such as injections and nerve blocks. These are nearly impossible to effectively blind. They also require a trained clinician and laboratory staff to deliver them, increasing the study costs and limiting the possible number of participants. On the other hand, there is a significant placebo effect in treating CRPS^22^, so an effort should be made to blind the study whenever possible.

The factors mentioned above lead to the low statistical power of conducted research and the poor quality of the existing data on CRPS treatment efficacy. This leads to a very limited choice of therapies and the lack of a scientifically proven “gold standard” to treat patients with CRPS. With some therapies, especially interventional ones, there is an uncertain risk-to-reward ratio, raising ethical concerns over offering them to desperate patients with chronic severe pain. Similar concerns can be raised about offering them sham interventions. This might be the reason for several studies not including a control group. To better assess the basis for said therapies, further research is required.

A requirement for high-quality research is having adequate tools. Over half of the papers reviewed mention difficulties in measuring outcomes with available assessment methods. These include pain interrupting assessments, the high subjectivity of pain assessments, roof effects for pain assessments, or missing data items. The lack of standardized, validated diagnostic methods is reflected in the large number of different tools used to assess CRPS. We counted a total of 99 measures across 55 studies, yet there was not a single one present in all the studies. The only assessment that was present in more than 50% of the papers was the Budapest Diagnostic Criteria, and this allows only for a dichotomous recognition of the presence or absence of CRPS, with no quantitative information. Attempts to ‘measure’ CRPS have been made in several dimensions, reflecting the complexity of the disorder. Unsurprisingly, the most common feature assessed was pain, mostly patient-reported, but also with some attempt at objectivity using the QST protocol. It is important to note that several studies independently tried to assess the impact CRPS and pain have had on patients’ lives, using a wide variety of questionnaires, with very little repetition. A similar dispersion of methods was seen across other analyzed methods, with few exceptions we would like to mention below. Six studies used the Tampa Scale for Kinesiophobia, which might be a new, interesting target in CRPS research. Several studies conducted motor, cognitive, and sensory functional assessments, looking to link function, rather than pain, with disease activity. It is worth mentioning that one study [36] used wrist accelerometry from a wearable device to assess global levels of activity. On the other hand, CRPS-specific tools did not find wider use (B-CRPS-BPDS—three, CSS—two, and ISS—two studies). All the above indicate the need for standardization in CRPS research, possibly with some new tools designed specifically for CRPS to assess disease activity and/or severity, which are easy to use in a clinical setting and do not require additional equipment. Improving the consistency of the assessment methods used has the potential to improve study comparability or maybe even allow for an international patient registry.

From the collected evidence, we can see that there are very few studies conducted in the field of CRPS; they mostly include small populations, hold low statistical power, and vary greatly in terms of methodology. The heterogeneity of the methods used suggests that none of them are effective in measuring CRPS activity and that we need guidelines for CRPS-related research.

With this in mind, we suggest that the following points be considered in future CRPS research:

1. Given patient heterogeneity, scarcity, and insufficient data on treatment efficacy, CRPS research should focus on small, high-quality studies, including case reports and n-of-1 studies, but with rigorous methodological detail.
2. Studies with experimental designs should include at least 30 patients with CRPS.
3. All studies should utilize the Budapest criteria, either clinical or research-based, to diagnose CRPS and explicitly state the criteria used.
4. Due to ethical concerns surrounding the administration of a placebo in the treatment of severe pain, study designs should favor head-to-head comparisons and avoid placebo-controlled designs.
5. Patients should be followed for at least 24 months to assess long-term treatment efficacy.
6. Disease activity measurements should be reproducible and include quantitative assessments of pain, anxiety, functional disability, quality of life, sleep quality, kinesiophobia, and cognition.

We also noted that many authors have addressed the issue we have encountered—the lack of a reliable method to assess CRPS activity. Pain alone is insufficient to monitor disease activity, as it often demonstrates a ceiling effect (with patients reporting maximal pain values) and low resolution (with many patients reporting minimal improvement in pain intensity, yet being able to resume some degree of their daily activities). Kinesiophobia may serve as a better parameter in this context, though it is more difficult to measure. Equipment-dependent methods offer objective measurements but are time-consuming and costly. We suggest the development of a new tool specifically designed to quantify CRPS activity. Based on our experience and the findings from our review, we propose the following key aspects for a potential CRPS activity index:

1. Ease of execution—The tool must be feasible in an outpatient setting, without the need for specialized equipment. It should focus on patient-reported outcomes through a self-reported questionnaire.
2. Brevity—It must be as concise as possible to increase the likelihood of widespread clinical adoption.
3. Simplicity—The tool must consist of straightforward tasks and questions. Patients often experience pain and may have cognitive impairments (due to pain, disease, or medication), which could hinder their understanding of more complex or abstract questions.
4. Multidimensionality—It should assess multiple aspects of the disease, including pain, anxiety, functional disability, quality of life, sleep quality, kinesiophobia, and cognition. It may also incorporate a graphic representation of the affected area.

### Limitations

The main limitation of the review is the small number of papers reviewed. This is mostly due to very few new studies being conducted in the field. We did not critically appraise the reviewed literature, as it is not required to do so in a scoping review, and we believe it would be counterproductive in the case of our study, where we are looking for potential drawbacks of studies. Another limitation was restricting the time to 2018 and onwards. This is the time period after the first core outcome measurement set for complex regional pain syndrome clinical studies (COMPACT) was published. We felt that extending the search further would make our findings less relevant to current practice. Finally, not all of the challenges we predicted emerged in the review, such as the lack of an animal model. We believe that these issues may not be as apparent from the perspective of clinical trials and could be explored further in future pre-clinical trial investigations.

## 6. Conclusions

Going back to the study goal, we can conclude that there is a wide variety of methods used to assess CRPS, but none of them are universal. On the other hand, authors tend to report similar issues, like low-quality data and the need for better assessment methods. Finally, we suggest that the standards for conducting CRPS research are developed as soon as possible. We still struggle to treat CRPS, with very few methods proven effective and very poor outcomes in long-lasting cases. A scientific framework will significantly improve the chance to bring the cure closer to our patients.

## Data Availability

No new data were created or analyzed in this study.
